# Prevention Aiming at Functioning—Describing Prevention in the Context of Rehabilitation: A Discussion Paper

**DOI:** 10.3390/ijerph20075399

**Published:** 2023-04-04

**Authors:** Christoph Gutenbrunner, Boya Nugraha, Thorsten Meyer

**Affiliations:** 1 Department of Rehabilitation and Sports Medicine, Hannover Medical School, 30625 Hannover, Germany; 2 Institute for Rehabilitation Medicine, Faculty of Medicine, Martin-Luther-University Halle-Wittenberg, 06112 Halle, Germany

**Keywords:** prevention, functioning, disability, International Classification of Functioning, Disability and Health (ICF), conceptualization, health strategy

## Abstract

The widely accepted model of prevention, including primary, secondary and tertiary prevention, focuses predominantly on diseases. The WHO provides a comprehensive model of health conceptualized on the basis of the International Classification of Functioning, Disability and Health (ICF). This paper develops a conceptual description of prevention aimed at functioning on the basis of the ICF model. Starting from the ICF-based conceptual descriptions of rehabilitation as a health strategy, a conceptual description of functioning prevention has been developed. Prevention aiming at functioning is the health strategy that applies approaches to avoid or reduce risks of impairing bodily functions and structures, activity limitations and participation restrictions; to strengthen the resources of the person; to optimize capacity and performance; to prevent impairments of bodily functions and structures; to prevent activity limitations and participation restrictions; to reduce contextual risk factors and barriers, including personal and environmental factors; to promote and strengthen contextual facilitators, with the goal of enabling people with impairments and people at risk of disability; and to maintain or improve the level of functioning in interactions with the environment. The proposed concept widens the scope of prevention to all aspects of functioning, including contextual factors.

## 1. Introduction

The association between prevention and rehabilitation is far from unequivocal. In terms of health strategies, they can be thought of as two qualitative approaches to health. At the same time, we could think of prevention and rehabilitation as two sides of the same coin, as gradual differences or even as parts of each other (e.g., in the concept of tertiary prevention). This ambiguity or even confusion represents the point of departure of this paper. From the perspective of rehabilitation, we will delineate the different ideas that contribute to this ambiguity and will come up with a proposal on how to conceptualize prevention with the context of rehabilitation.

Rehabilitation has been conceptually described as a health strategy that is based on the World Health Organization’s (WHO’s) comprehensive model of the International Classification of Functioning, Disability and Health (ICF) [1,2]. The ICF model defines the functioning of an individual with a health condition as a positive interaction with the environment. In this context, functioning comprises bodily functions and structures, activities and participation [1]. Contextual factors, i.e., personal factors and environmental factors are included in the model as barriers or facilitators of functioning. Against this background, the aim of rehabilitation is conceptually described as optimizing the functioning of individuals in need. This goal is achieved through approaches to assess functioning, to optimize a person’s capacity and to develop a person’s performance and through other approaches [2]. Rehabilitation focuses on individuals and includes the provision of a facilitating environment as another relevant approach of the rehabilitation strategy [2]. On the contrary, interventions at the population or community level, such as community development programs [3] or the universal design approach [4], are considered public health interventions [5]. Last but not least, the conceptual description of rehabilitation as a health strategy includes approaches to strengthen the resources of the person.

As rehabilitation targets individuals, the underlying concept of functioning should be reflected in the context of the WHO’s understanding of health. Already in its constitution, the WHO has defined health as “a state of complete physical, mental and social wellbeing and not merely the absence of disease or infirmity” [6]. This means that, according to the WHO, health consists of two components:

(1) The absence of disease.
(2) Physical, mental and social wellbeing.

The absence of disease is medically defined as a physical and mental state where no disease symptoms are present and no pathology can be medically detected. However, defining the wellbeing component is much more complex. Stucki et al. [7] proposed to operationalize health on the basis of the International Classification of Functioning, Disability and Health [1]. One key to understanding health within this model is the ICF construct of *functioning*. As mentioned above, functioning is the result of the interaction of a person with a health condition and contextual factors and refers to the lived experience of a person [7]. However, contrary to the disease concept, functioning is a positive concept.

Prevention and health promotion can be seen as main health strategies, along with treatment, rehabilitation and palliative strategies [8]. Prevention has also been integrated into the concept of universal health coverage (UHC), which means that health promotion and prevention should be available for all [6].

**Figure 1:**
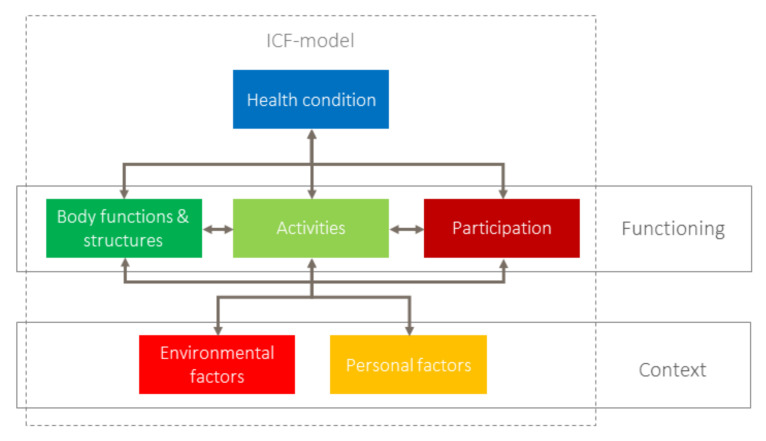
International Classification of Functioning, Health and Disability (ICF) model [1] (Reprinted/adapted with permission from [1]. Copyright year 2001, copyright owner’s WHO”).

According to the most widely used concepts of prevention, classified into primary, secondary and tertiary prevention, prevention aims to combat diseases or disorders through early detection, reducing risk factors and slowing down progression [9]. It also includes reducing the harm originating from a disease, addressed as tertiary prevention (see below). This stepwise approach is based on a linear model of the development and progression of diseases that corresponds to the model of the International Classification if Impairments, Disability and Handicap (ICIDH). However, the ICIDH model has been replaced by the ICF model for good reasons. The former’s linearity implies a unidirectional process from disease to handicap and lacks the societal component of disability [10]. ICF, on the contrary, integrates more-complex, bi- and multidirectional interactions among its components (Figure 1).

As mentioned above, rehabilitation also includes interventions that target strengthening the resources of a person [2]. This not only includes strengthening coping or resilience but also may include the prevention of alterations of bodily structures (e.g., prevention of pressure sores) and functions (e.g., prevention of contractures). It also includes activities (e.g., preventing loss of activity level) and participation (including quitting a job). This type of rehabilitation goes beyond the “classical” concepts of prevention that focus on diseases only. Against this background, this paper analyzes the existing definitions of prevention in the light of the WHO’s definition of health and develops a proposal to conceptually describe prevention in the context of functioning in light of the WHO’s comprehensive model of functioning, which should be distinguished from prevention targeting (the development of) diseases.

### 1.1. Conceptualizations of Disease Prevention

The basic concept of prevention originated from the 1950s and became an emerging concept as a pathophysiological understanding of the mechanism of diseases was elucidated [11]. One main stream of disease prevention originated from the prevention of infectious diseases, e.g., by avoiding infection via hygiene measures [12]. Later on, as the relevant factors for the development of chronic diseases were discovered, the scope of prevention has been extended (e.g., to nutritional risk factors for metabolic diseases such as diabetes and hyperuricemia or behavioral risk factors for hypertension and myocardial infarction) [13].

The concept of prevention was systematically described in the 1950s in the United States by the Commission on Chronic Illness [14,15,16]. Definitions of prevention focus on diseases (and disease symptoms). Although there are inconsistencies within the definitions and descriptions of the levels of prevention [17], three main strategies of prevention prevail in the literature (two additional concepts of prevention have appeared in the literature but have not been widely acknowledged: (1) *Primordial prevention* is considered the most early type of prevention, as it means to avoid the occurrence of risk factors of diseases in the first place, e.g. supporting exercise and mobility in preschool children to prevent overweight or obesity as a risk factor for cardiovascular diseases [18]. The delimitation from primary prevention or health promotion is not clearcut. (2) The term *quaternary prevention* was introduced to characterize “action taken to protect individuals from medical interventions that are likely to cause more harm than good” [19] (page 109)):

- *Primary prevention*: “Methods to avoid occurrence of disease either through eliminating disease agents or increasing resistance to disease. Examples include immunization against disease, maintaining a healthy diet and exercise regimen, and avoiding smoking” [20].
- *Secondary prevention*: “Methods to detect and address an existing disease prior to the appearance of symptoms. Examples include treatment of hypertension (a risk factor for many cardiovascular diseases) and cancer screenings” [20].

Following this line of thought, *tertiary prevention* deals with clinically manifestant diseases [20]. On the one hand, diseases should be treated in order to avoid their progression or to prevent disease-related incidences, such as myocardial infarction. On the other hand, consequences of the diseases itself or of its treatment, such as pressure ulcers for patients having to stay in bed or people using wheelchairs should be prevented through appropriate measures. Both approaches may contribute to reducing the disabilities seen as consequences of a given disease or health condition (together with death).

Sometimes, rehabilitation and tertiary prevention have been used synonymously, which does not take into account paradigm changes in rehabilitation owing to the introduction of the ICF and which is therefore incorrect [21]. In addition, the use of the term *prevention* is restricted mainly to the concepts of primary and secondary prevention (and also health promotion), e.g., in prevention textbooks or compilations of prevention programs [21].

As mentioned before, the stepwise linear approach of prevention (from primary to secondary to tertiary prevention) is mirrored in the definition of handicap and disability from the ICIDH (from impairment to disability to handicap). Both are grounded on the assumption of linear processes. The ICIDH has been replaced by the ICF, which provides a model of functioning. This model takes into account multiple interactions between its components: activities and participation also can modify bodily functions and people’s health conditions. It seems a worthwhile endeavor to analyze the degree to which this change of perspective, which has characterized the field of rehabilitation, is reflected in prevention concepts.

### 1.2. Health Promotion

The definition of *health promotion* is much broader than that of *prevention*. It can be seen as *public health strategy* and does not target on the individual [21]. According to the WHO, it can be defined as follows:

“Health promotion is the process of enabling people to increase control over, and to improve, their health. It moves beyond a focus on individual behavior toward a wide range of social and environmental interventions. As a core function of public health, health promotion supports governments, communities and individuals to cope with and address health challenges. This is accomplished by building healthy public policies, creating supportive environments, and strengthening community action and personal skills” [22].

Thus, health promotion includes some components of the ICF (e.g., environment) and quality of life. It can be linked to the philosophy of the ICF and the recent conceptualizations of health [7].

In the 1986 WHO Ottawa Charter for Health Promotion [23], health “a resource for everyday life, not the objective of living. Health is a positive concept emphasizing social and personal resources, as well as physical capacities. Therefore, health promotion is not just the responsibility of the health sector, but goes beyond healthy life-styles to well-being”. Thus, while health promotion and rehabilitation have a common emphasis on resource orientation, a positive health concept and the explicit integration of environmental factors, they substantially differ in the addressee: population (sub)groups on the one side and individuals with (imminent) functioning limitations on the other side.

### 1.3. Need for Conceptualization of Prevention Aiming at Functioning

Given the enormous relevance of disease prevention, against the background of the WHO’s definition of health prevention must include a broader perspective integrating medical, social and quality-of-life aspects of health. The International Classification of Functioning, Disability and Health [1] provides a framework to operationalize the lived experiences of persons with health conditions [7]. The most important premise is that functioning limitations or disability, respectively, occur from the negative interaction between a person with a health condition and their environment [1]. Thus, the character and intensity of the disease are not the only factors or even the main factor for experiencing disability. Activities, participation and both personal and environmental factors are also crucial.

In rehabilitation, this perspective has become crucial. Additionally, as preventive interventions have always been integrated into rehabilitation practice, rehabilitation and prevention could profit from a broader concept of prevention that explicitly aims at functioning in addition to disease prevention [21].

Another argument for a conceptualization of prevention aiming at functioning comes from the World Report on Disability (WRD) [5]. The WRD underlines the necessity of dealing with aspects of preventing the loss of functioning: “Preventing disability should be regarded as a multidimensional strategy that includes prevention of disabling barriers as well as prevention and treatment of underlying health conditions” (page 8) [5]. Additionally, the WRD stresses that “rehabilitation measures target bodily functions and structures, activities and participation, environmental factors, and personal factors. They contribute to a person achieving and maintaining optimal functioning using the following broad outcomes: prevention of the loss of function, slowing the rate of loss of function, improvement or restoration of function, compensation for lost function maintenance of current function” (page 95–96) [5].

A fundamental reflection that led to developing a conceptualization of prevention in the context of rehabilitation was the fact that prevention, in addition to its sole focus on diseases, is always meant to occur *before* an incident or specific phase of the course of a disease. Additionally, the prevention concepts from the perspective of public health do not primarily take the perspective of the individual but instead are defined as intervention at a population level [24], while a more clinical view of prevention, often related to secondary prevention, is also focused on the individual patient. On the contrary, rehabilitation also intervenes when a certain health and functioning status has already developed. That means that rehabilitation approaches take place *during* and *after* an incident or the course of the disease. Rehabilitation targets, as mentioned above, the individual and their lived experience [25]. Furthermore, primary prevention always aims at avoiding an incident or risk factors for the development of a disease, whereas rehabilitation includes the concept of empowering and helping a person and includes facilitators for optimal functioning.

This article aims at developing an ICF-based conceptual description of prevention aiming at functioning. The article takes a theoretical approach to conceptualize prevention in the context of the rehabilitation strategy. We use the term *prevention* here in a broader sense, comprising also aspects of salutogenetic or resource-oriented approaches conceptually related to health promotion. As a conceptual approach, the article aims not to produce evidence-based knowledge but rather to produce theory-based knowledge [26].

## 2. Materials and Methods

In order to develop a theoretical concept to describe rehabilitation approaches aiming at improving individual levels of functioning and to prevent the loss of functioning, a methodological approach was chosen, one that has already been used to develop conceptual descriptions of rehabilitation as a health strategy [2], of physical and rehabilitation medicine (PRM) [27] and of rehabilitation services [28]. This methodology is based on the framework of the International Classification of Functioning, Disability and Health (ICF; WHO), which comprehensively describes all the relevant domains of functioning and at the same time provides a well-accepted framework of functioning and disability. The structure is a one-sentence description: it starts with the basis or context; is followed by the means or approaches and the target groups; and ends up at the overall goal of the strategy. Where appropriate, the terminology of the ICF has been used.

On the basis of a literature search and a first proposal, an iterative discussion process among the authors was used. The intermediate results were presented and discussed in a number of national and international congresses for rehabilitation sciences (German rehabilitation science congresses) and for physical and rehabilitation medicine (international congresses on PRM). The results of this process have been published as a discussion paper, with the goal of receiving comments and further input.

## 3. Results

As mentioned before, the proposal for a conceptual description of prevention aiming at functioning (Table 1) is related to the conceptual descriptions of rehabilitation as a health strategy [2] and the conceptual description of physical and rehabilitation medicine [27]. Both of these descriptions have been approved by the European and international PRM bodies and are in line with the recent WHO definitions of rehabilitation. Another application of an ICF-based conceptual description has been that of rehabilitation services [28], which was the basis of the development of the International Classification of Service Organization in Rehabilitation (ICSO-R) [29,30].

The perspective of the abovementioned conceptual descriptions is that they use the comprehensive WHO model of functioning as a basis and common ground of the description. They also describe measures (interventions) as integral parts of activities and sectors or services that are needed to apply the interventions. All the conceptual descriptions end up with the following: “to enable persons with health conditions experiencing or likely to experience disability to achieve and maintain optimal functioning in interaction with their environment”.

As the conceptual description of prevention aiming at functioning has been developed in the context of rehabilitation medicine, at this stage, it is defined only for this context. Future discussion will show whether this also applies to other contexts (education, social welfare and others). Another prerequisite is the underlying comprehensive model of functioning. The approaches include risk assessment, resource strengthening, training of capacity and performance, prevention of impairments, activity limitations, participation restrictions and the reduction of contextual risk factors, i.e., environmental barriers. As it is a preventive strategy, the strengthening of facilitators from the ICF domains of contextual factors are also part of it. Different from the other conceptual descriptions, two additional explanations are added: one is to clarify that functioning prevention comprises interventions at the individual’s level, including the person’s context (personal factors and their environment). The second one highlights that prevention aiming at functioning is not restricted to a specific setting, but rather, it may be part of all levels of healthcare and along the continuum of care.

The goals of “maintaining the level of functioning” directly correspond to the goal of rehabilitation, which aims at “achieving optimal functioning”. The concept of prevention aiming at functioning adds to the target groups of rehabilitation the group of persons with impairments and at risk of developing a disability. The overlap of both approaches is the reason why the conceptual description of prevention aiming at functioning is related to the context of rehabilitation. However, the authors are convinced that a separate (or additional) conceptual description is warranted.

We provide some examples for prevention aiming at functioning in rehabilitation medicine and beyond. From the point of view of a clinician, performing prevention aiming at functioning is a core element of rehabilitation medicine and in most cases an integral part of rehabilitation. Some clinical examples are as follows:

- In early rehabilitation, the prevention of contractures, the loss of muscle function and other preventive measures are important for good long-term functioning outcomes (including participation). Another component is early mobilization (as component of activities).
- In chronic diseases (e.g., in mental disease), the promotion of activities and participation as early as possible may limit the development of the experience of disability.
- The early detection of impairments of bodily functions or structures in newborns and children (e.g., in the field of communication) can lead to preventing a vicious circle of delayed development.
- Removing barriers from the environment of the individual and optimally turning them around into facilitators (e.g., wheelchair-accessible apartment, workplace adaptation, the provision of appropriate assistive technology, and providing information to relatives).

A broader concept of prevention aiming at functioning can be addressed as part of so-called prehabilitation [31]. This includes measures to prevent sequelae of medical interventions such as surgery, cytostatic medication, radiation and immobilization. Examples of such interventions are presurgical muscle training or the training of respiration techniques, aerobic exercise during cancer treatment to reduce side effects, motor training for transfers or using crutches and other devices needed after surgery [32].

Beyond rehabilitation, it seems to be important to describe other approaches to prevention aiming at functioning that are not related to any emerging health condition or pathology but that have already started with health promotion in the situation of “primary prevention”. This level of prevention defines (and evaluates) interventions that may help people without any impairment to avoid developing an impairment or disability. Such interventions might focus on the individual level (e.g., physical training to strengthen bodily functions or mental training to strengthen resilience) or at the level of environmental factors (e.g., creating barrier-free environments or supporting inclusive attitudes in society) [8,33].

## 4. Discussion

Jamoulle et al. [17] analyzed the definitions and operationalization of prevention and found out that there are large differences in the descriptions of primary, secondary, and tertiary prevention. They also pointed out that the linearity of the underlying understanding, historically coming from the prevention and management of infectious diseases, did not reflect the reality of prevention in the context of healthcare, which has to consider many interactions, e.g., in the situation of multimorbidity and the potential prevention of chronic diseases [17].

Starfield et al. [34] stressed that focusing on individual prevention in health matters (in the sense of the absence of disease) fails to include societal aspects of prevention. They proposed to include a societal level that includes environmental planning, public advocacy, resource mobilization, information systems and others in addition to the individual level of prevention. They also point out that the burden of disability should be in the focus of prevention rather than the burden of disease [34].

Another approach toward a broader perspective comes from the management of occupational back pain [35]. Starting from the relevance of contextual factors that influence the prevalence and consequences of back pain, Loisel et al. addressed factors in the workplace system, the compensatory system, the healthcare system and personal life within a prevention strategy. They named this concept “disability prevention” [35], but the authors did not develop a more generalized and comprehensive model for the prevention of disability.

The prevention of disability is also addressed in geriatric medicine. Luukinen et al. [36] published a paper on the effect of exercise on the development of disability. It is a classical RCT using functioning parameters as outcomes and does not reflect the complexity of the interaction of individuals with the environment according to the ICF model.

Our conceptional description of prevention aiming at functioning seems to be able to provide a common framework and terminology for these different approaches.

With regard to health promotion, Rimmer [37] pointed out that health promotion for persons with disabilities is a neglected field. He stated not only that this is relevant for the reduction of secondary conditions but that it must include the maintenance of functional independence, to enable participation in leisure and enjoyment and to enhance people’s overall quality of life [37]. Another author also stressed the relevance of reducing environmental barriers to good health [38]. This approach coincides in many aspects with our conceptual reflections. Some trials have shown that specific programs with this purpose may lead to favorable health and quality-of-life outcomes [39,40].

To the best of our knowledge, a comprehensive model of prevention based on the WHO’s model of functioning, disability and health has not yet been published. Our approach aims to contribute to the theoretical basis of rehabilitation. It is therefore an approach to generate new theory-based knowledge [26] that might contribute to scientific research in the field of health systems and services as well as to systematically describe and evaluate preventive approaches.

The herein provided conceptual description of prevention aiming at functioning is based on the previous work of conceptually describing rehabilitation [2], PRM [27] and rehabilitation services [28]. It is a one-sentence description including a background model, (interventional) factors and the goal of the described strategy, intervention or organization. Using such an established approach makes the problem manageable and the results comparable to other conceptual descriptions. It also shows the overlaps that are obvious, in particular with regard to the following goal: “to enable persons with health conditions experiencing or likely to experience disability to achieve and maintain optimal functioning in interaction with their environment”. Another strength of this approach is that it is based on the well-accepted model of functioning, disability and health [1]. The other conceptual descriptions have been accepted by a larger scientific community [2,27] and have proven useful to derive more-detailed operationalizations, such as the International Classification of Service Organization in Rehabilitation (ICSO-R 2.0) [30].

With regard to the content, our approach closes the gap in the classical model of primary, secondary and tertiary prevention. It allows a broader perspective of health, including societal participation, wellbeing and quality of life. It also respects the fact that disability is not an attribute of a person but rather occurs only in interactions with the environment [5]. It also allows for taking contextual factors, such as the environment and personal factors, into consideration and thus includes them in a health-related prevention model.

As mentioned above, rehabilitation has been conceptually described as the health strategy that diagnoses the level of functioning and applies interventions “to enable people with health conditions experiencing or likely to experience disability to achieve and maintain optimal functioning in interaction with the environment” [2]. Within this context, rehabilitation has (or must have) a preventive component too: the prevention of impairments and disabilities, including functional and structural impairment, activity limitations and participation restrictions. Such preventive measures must be addressed as early as possible and not only after a disease has led to the (significant) loss of functioning. Thus, also here, the aspects of strengthening functioning, the early detection of disability (and/or the risk of developing disability) and the reduction of disability must be addressed (the latter in rehabilitation).

The proposed conceptual description of prevention aiming at functioning overlaps with the concept of health promotion. Health promotion also includes the empowerment of people or the strengthening of resources, as well as behavioral aspects and the activities of society (e.g., at the community level) [5]. It includes strategies to reduce tobacco and alcohol abuse and promotes physical activity, healthy diets and also mental health. It also addresses societal attitudes and behaviors, such as domestic abuse, public awareness and problems occurring from legislation [5]. However, a specific focus on disability is also lacking here, e.g.., one that includes supporting diversity, reducing negative attitudes toward persons with disabilities and humanmade barriers, strengthening coping strategies and solving health and societal problems.

The naming of the concept of rehabilitation aiming at functioning has some relevant inherent problems too. In the ICF, *disability* is defined as problems or limitations of functioning. *Disability* is the negative and *functioning* the positive aspect of interactions between a person with a health condition (and impairment) and their environment [1]. *Rehabilitation* should also defined according to its potential to reduce disability and improve functioning [2]. However, such an approach has been criticized by an organization of persons with disabilities. The main argument is that being a “person with a disability” does not necessarily mean that the person needs rehabilitation (or “to be rehabilitated”). Of course, this also meets with a possible term such as “disability prevention”. However, in the ICF context and along the axis of “functioning and disability”, this would be the correct term as the lived experience of disability is the status that needs to be prevented. This also disrupts the fundamental principle of shared decision-making in partnerships between the person and the provider [2], which should respect the principle of autonomy for patients with disabilities, according to the UN convention on the rights of people with disabilities. However, given the arguments from the organization of persons with disabilities, the authors decided not to use this term.

Another reflection was to use the term *dys-functioning*, which theoretically meets the point and includes the target to “optimize functioning” [2]. However, the term of *dys-functioning* has not been introduced within a medical context; it remains in technical sciences and engineering. In this situation, the authors concluded that the term *prevention aiming at functioning* was the most neutral term and at the same time described the content well.

The major limitations of the approach of this paper are as follows: it is not based on a broader consensus, e.g., within scientific societies or other groups of experts, and it does not contain a proof of concept from analyzing successful prevention programs aiming at improving functioning and reducing disability. Another open point is the borderline between “prevention aiming at functioning” and other “functioning interventions” as used in rehabilitation. Here, the general dilemma arises when we talk about health strategies or interventions. This is very similar to the rehabilitation definitions that range from “rehabilitation is a health strategy” to “rehabilitation is a set of interventions”. Most likely, this dilemma cannot be solved, because both aspects cover important aspects of rehabilitation and/or prevention. Furthermore, clear distinctions into mutually exclusive categories between functioning prevention and rehabilitation cannot be drawn. However, this problem is obvious in other definitions of health strategies and interventions, e.g., for treatment and rehabilitation. From the perspective of rehabilitation medicine, we could argue, in line with [41], that interventions in most cases include preventive, curative and rehabilitative aspects, which lead to their calling rehabilitation medicine the “medicine of functioning”.

## 5. Conclusions

The proposed concept of prevention aiming at functioning, based on the framework of the International Classification of Functioning, Disability and Health, includes a broader understanding of health as it has been defined by the WHO [1]. It is based on the conceptual descriptions of the rehabilitation strategy, enhances the scope of all aspects of functioning (bodily functions, activities, and participation) and includes contextual factors (personal and environmental factors). The model is closely connected to the rehabilitation approach but also reflects relevant aspects of health promotion. The authors are convinced that the proposed model will contribute to an understanding of how to prevent disabilities and may be used to develop innovative prevention concepts. However, the authors are aware that a broader and deeper discussion is needed, one in which studies must show the relevance of prevention.

## 6. Outlook

As this paper consists only of conceptual reflections, a broader discussion within the scientific community and a proof of concept is needed. For that reason, the authors would be happy to initiate ongoing discussion. This will give an opportunity to add another concept to the understanding of prevention with regard to the development of disability or, in other words, to conceptualize prevention in light of rehabilitation.

## Figures and Tables

**Table 1:** Proposal for a conceptual description of prevention aiming at functioning.

**Line**	**Text**
**Conceptual Description:**	
**In the context of rehabilitation**	
and based on the WHO’s **comprehensive model of functioning,**	
**prevention aiming at functioning** is the **health strategy** that applies approaches	
to **assess the risks** of impairments of bodily functions and structures, activity limitations and participation restrictions;	
to strengthen the **resources of the person;**	
to optimize **capacity and performance;**	
to **prevent impairments** of bodily functions and structures;	
to **prevent activity limitations** and **participation restrictions;**	
to **reduce contextual risk factors and barriers**, including personal and environmental factors;	
to **promote and strengthen contextual facilitators.**	
Its goal is to avoid and reduce the risks of impairments and functioning limitations and to enable persons with impairments at risk of disability and persons experiencing disability to **maintain or improve their level of functioning** in interaction with the environment.	
**Additional explanations:**	
This goal can be achieved by **functioning interventions at the person’s level**, including protection against detrimental environmental factors for the individual.	
**Functioning interventions** can be applied **along and across the continuum of care** (*including prehabilitation, acute, postacute and long-term care*), in **different settings** (*including hospitals, rehabilitation facilities and the community*), **at all levels of healthcare** (*including primary, secondary and tertiary care*).	

## Data Availability

Not applicable.
